# Differentiating adults who think about self-harm from those who engage in self-harm: the role of volitional alcohol factors

**DOI:** 10.1186/s12888-019-2292-3

**Published:** 2019-10-28

**Authors:** Ambrose J. Melson, Rory C. O’Connor

**Affiliations:** Suicidal Behaviour Research Laboratory, Institute of Health & Wellbeing, University of Glasgow, Glasgow, UK

**Keywords:** Self-harm, Suicide, Ideation-to-action, Alcohol

## Abstract

**Background:**

Self-harm, an act of self-poisoning or self-injury irrespective of motivation, is a major public health concern. Use of alcohol prior to or alongside acts of self-harm is common but little is known about the alcohol-related mechanisms of self-harm enaction. We utilised an ideation-to-action approach to clarify the extent to which volitional alcohol factors differentiated those who have thoughts of self-harm but do not act on them (self-harm ideation) and those who engage in self-harm (self-harm enaction).

**Methods:**

Cross-sectional analyses of the baseline phase of the Health Lifestyle and Wellbeing study: 1546 adults (1079 female; Mean age = 34 y; 92% White) resident in Scotland completed measures of demographics, lifetime self-harm, volitional alcohol factors and psychosocial factors. Multinomial logistic regression compared those with a history of self-harm thoughts (‘ideation’, *n* = 297), self-harm acts (‘enaction’, *n* = 346) and ‘controls’ (*n* = 897) to identify volitional alcohol factors associated with self-harm enaction.

**Results:**

Volitional alcohol factors differentiated those with a history of self-harm enaction from those with a history of self-harm ideation (as well as those with no history) in initial models adjusted for demographics and depressive symptoms: the self-harm enaction group reported stronger alcohol-related negative urgency (OR = 1.74, 95% CI 1.41–2.16, *p* < .001), more frequent heavy drinking (OR = 1.46, 95% CI 1.24–1.72, *p* < .001) and stronger expectancies that drinking alcohol leads to negative self-perceptions (OR = 1.33, 95% CI 1.03–1.72, *p* = 0.03) and markers of self-harm risk (OR = 1.64, 95% CI 1.18–2.30, *p* = 0.004). Alcohol-related negative urgency and heavy-drinking frequency continued to differentiate those in the self-harm enaction group from those in ideation group in multivariate models. Consistent with theoretical models positing phase-specific moderators of self-harm ideation and enaction, psychosocial factors (perceived stress, support, negative mood regulation expectancies) differentiated those with a history of self-harm ideation from those without but not those in the ideation and enaction groups.

**Conclusions:**

Management of self-harm risk requires better understanding of alcohol-related mechanisms of self-harm enaction. Volitional alcohol factors may play a role in governing the translation of self-harm thoughts into self-harm acts.

## Background

Self-harm refers to any act of self-poisoning or self-injury irrespective of motivation [1] and is a major public health concern [2]. The aetiology is complex and self-harm is thought to arise through an interplay of genetic, biological, psychiatric, psychological, social and cultural factors [3, 4]. Among many known risk factors for self-harm [4], alcohol is estimated to be involved in 20% of observed global morbidity and mortality attributable to self-harm [5]. In the UK more than half of individuals admitted to emergency departments following self-harm will have consumed alcohol alongside or in the hours preceding the episode [6]. The status of alcohol as a major risk-factor for self-harm is well-recognised, with research activity often centred upon the role of alcohol-use disorders (AUDs; e.g., harmful use, dependence) [7, 8] as diagnostic categories conferring an increased risk for self-harm and suicide (e.g., [9, 10]). This has led to advances in our understanding of treatment and management of self-harm risk. However, we need to move beyond diagnostic categories of AUD to elucidate the alcohol-related mechanisms of self-harm enaction.

Although functions and motives for engaging in self-harm are varied and complex, for many people self-harm is a response to distressing internal emotional and cognitive states [11–17]. Immediate experiences of pain and suffering may escalate when alcohol-induced cognitive and perceptual ‘myopia’ narrow attention to the proximal causes of distress whilst also limiting one’s capacity to identify adaptive coping strategies [18]. To the extent that intoxication facilitates tunnel vision of one’s acute distress, self-harm enaction may increasingly be seen as the salient response [19, 20]. Furthermore, when faced with heightened negative emotion, those with a behavioural propensity to engage in impulsive acts in order to reduce negative affect may also be at elevated risk of engaging in self-harm [21–23]. Because any tendency to engage in rash actions in response to distress may be amplified following alcohol use, drinking episodes are likely to be a time of acute risk for self-harm enaction. Alcohol expectancies, representing beliefs about the effects of drinking alcohol, are proximal determinants of alcohol-related behaviour and outcomes, with belief in expected drinking outcomes directing future actions [24, 25]. Alcohol expectancies therefore represent additional mechanisms of self-harm enaction if the anticipated outcomes of drinking trigger a transition from thinking about self-harm to self-harm acts [19, 26, 27]. Expecting alcohol to bolster confidence (i.e. “liquid courage”) or facilitate risk-taking and aggressive behaviour, for example, may represent specific expected outcomes of drinking which override protective barriers such as fear and pain [19, 26, 27].

Research on alcohol-related mechanisms that contribute to self-harm enaction will enhance understanding of the aetiology and management of self-harm. This focus is consistent with ideation-to-action approaches, which have improved our ability to distinguish between individuals who think about self-harm and those who act on their thoughts [14, 28–31]. In particular, the Integrated Motivational-Volitional (IMV) model provides an explicit theoretical basis for integrating and sequencing factors and constructs associated with the emergence of thoughts of self-harm (i.e. ‘pre-motivational/motivational phase factors’) as well as those which govern the transition from thinking about self-harm to acting on those thoughts (i.e. ‘volitional phase factors’) [30, 32]. In the present study, within the context of the IMV model, we investigated several alcohol factors, each representing a distinct possible path from self-harm thoughts to self-harm enaction. Given that alcohol use is highly prevalent within the general adult population of Scotland [33], we investigated these factors in a community sample of adults with and without a history of thinking about or engaging in self-harm. Guided by the IMV model [30] we expected drinking to intoxication, a tendency to engage in rash acts in order to alleviate negative affect when drinking, and alcohol expectancies, to act as volitional phase alcohol factors; thereby being particularly associated with self-harm acts. We therefore hypothesised that, in multivariate models, (i) those who have acted on their thoughts would differ significantly from those who have thoughts of self-harm (but have not acted upon these) and those with no history of thoughts or acts on each of these volitional phase alcohol factors. We also hypothesised that (ii) those who had thoughts about or engaged in self-harm would differ significantly from controls on a range of pre-motivational/motivational phase factors (perceived stress, social support, optimism and expectancies for negative mood regulation) but there would be no difference between those with thoughts of self-harm and those who have enacted self-harm.

## Methods

### Participants and procedures

Throughout 2015, adults aged 18 years and over residing in Scotland were invited to participate in the Health, Lifestyle and Wellbeing Study, a prospective online study addressing the interplay of lifestyle behaviours with physical and psychological health, morbidity and wellbeing in the Scottish adult population. Participants were recruited via places of employment, education and community networks including large commercial and local authority employers, university registers, online community groups and forums. Recruitment invitations provided information about the aims and nature of the study, a survey URL and contact details for the research team. The information provided also stressed the voluntary nature of participation and independence of the research team from employers or education institutions. Written informed consent was obtained from all participants and study procedures complied fully with the Helsinki Declaration of 2008. Local approval was provided by the College of Medical, Veterinary and Life Sciences Ethics Committee at the University of Glasgow (project no: 200140114). The questionnaire required 15–20 min to complete and included questions on a range of socio-demographic, lifestyle, mental and physical health status as well as psychological measures. Entry into a prize draw was offered as compensation for participation time. Only the measures relevant to the present study are reported here. The present research reports cross-sectional baseline findings from an initial study sample of 1546 adults (1079 female, 460 male; 7 did not provide information on gender), aged on average 34.39 years (SD = 13.03; range 17–69), who were predominantly White (91.8%), with the majority well-educated (58.6% degree or post-graduate qualification) and in employment (65.3%). Given that a wide range of recruitment methods were used in the study, and that interested individuals self-selected to participate, response rates are not known. Further information on recruitment and participation can be found in Additional file 1 (Figure S1).

### Measures

#### Outcome

Self-harm history (‘enaction’ vs. ‘ideation’ vs. ‘control’) was ascertained using items adapted from the Adult Psychiatric Morbidity Survey and Child and Adolescent Self-harm in Europe Survey [34, 35]. The self-harm enaction group comprised individuals who responded ‘yes’ to ‘Have you ever made an attempt to take your life, by taking an overdose of tablets or in some other way?’ or ‘Have you ever deliberately harmed yourself in any way (but without wanting to kill yourself)?’. The ideation group included individuals who responded ‘no’ to the enaction questions and ‘yes’ to ‘Have you ever seriously thought of taking your life, but not actually attempted to do so?’ or ‘Have you ever seriously thought about trying to deliberately harm yourself (without wanting to kill yourself) but not actually done so?’. The individuals in the control group responded ‘no’ to each item. This classification is based on the National Institute for Health and Care Excellence [1] definition of self-harm which incorporates self-harm irrespective of motive (i.e., including suicide attempts and non-suicidal self-harm).

#### Covariates

The 20-item Centre for Epidemiologic Studies Depression Scale-Revised (CESD-R; [36]) was included as a valid measure of depressive symptoms (e.g., worthlessness, fatigue, appetite loss) in community populations [37]. In the present study internal consistency^1^ was excellent (α = 0.93). Age and gender were also recorded.

#### Pre-motivational/motivational phase factors

Recent perceived stress was assessed using the Perceived Stress Scale Short Form (PSS-S; [38]), a reliable and valid 4-item scale containing items such as ‘in the past four weeks, how often have you felt difficulties were piling up so high that you could not overcome them?’ [38, 39]. Negative mood regulation expectancies, reflecting subjective beliefs in ability to alleviate negative mood states, were assessed using the reliable and valid 30-item Generalised Expectancies for Negative Mood Regulation Scale (NMR; [40, 41]) which includes items such as ‘When I’m upset, I believe that I can usually find some way to help myself feel better’. Optimism and social support were measured using items from the Warwick-Edinburgh Mental Well-being Scale [42] (optimism: ‘I’ve been feeling optimistic about the future’; social support: I‘ve been feeling close to other people’, ‘I’ve been feeling loved’). In the present study internal consistency of the multi-item scales assessing pre-motivational/motivational phase factors ranged from good to excellent (α’s 0.80–0.91).

#### Volitional phase alcohol factors

The first volitional phase alcohol factor, drinking to intoxication, was assessed using the frequency of heavy drinking item from the Alcohol Use Disorders Identification Test [43, 44]. Individuals reported how frequently they consumed 8 or more UK units (≥64 g) of alcohol on a single occasion over the past 6 months. Negative urgency is the tendency to engage in rash actions in order to reduce negative affect [21, 22]. In the present study, the 12-item Negative Urgency subscale of the UPPS Impulsive Behaviour Scale [23, 45] was modified to make the tendency to engage in rash actions in response to negative affect contingent on drinking episodes. Standard negative urgency items such as ‘when I am upset I often act without thinking’ were therefore modified to ‘when I am upset and I drink alcohol, I often act without thinking’ in order to create an alcohol-related negative urgency scale (Negative Urgency-A). Internal consistency of the 12-item Negative Urgency-A scale was excellent (α = 0.94).

Alcohol expectancies were assessed using six of the seven^2^ subscales comprising the Comprehensive Effects of Alcohol Questionnaire (CEOA; [46]) as well as a number of novel expectancy items that we generated. The original CEOA questionnaire is a reliable and valid measure of alcohol-contingent expectancy beliefs covering different alcohol expectancy outcome domains [46–48]. Representative items for the six CEOA subscales used in this research include ‘when I drink alcohol, I expect that I [‘would be outgoing’ (Sociability), [‘would feel calm’] (Tension Reduction), [‘would feel unafraid’] (Liquid Courage), [‘would have difficulty thinking’] (Cognitive and Behavioural Impairment), [‘would feel self-critical’] (Self-Perception) and [‘would take risks’] (Risk and Aggression) [46]. Internal consistency of the CEOA subscales ranged from adequate to excellent (α’s 0.73–0.90). In addition to the CEOA subscales, based on psychological theory [30, 49], seven novel items targeting alcohol expectancies for specific markers of self-harm risk were also included. This Self-Harm Alcohol Expectancy Scale comprised seven items based on the standard expectancy item structure: ‘when I drink alcohol, I expect that I [‘would feel disconnected’], [‘feel alone’], [‘feel defeated’], [‘feel hopeless’], [‘feel trapped’], [‘think about suicide’], [‘attempt suicide’]. Internal consistency of the novel Self-Harm Alcohol Expectancy Scale was also good (α = 0.82).

### Statistical analysis

All analyses were conducted using SPSS version 24 [50]. Missing data were observed for 0.6–10.9% of data across study measures. We used missing at random (MAR) procedures based on multiple imputation (*n* = 20 iterations) [51] to impute missing data for continuous-level measures only. Further information can be found in Additional file 1 (Figure S1). Planned tests of study hypotheses involved univariate multinomial logistic regression models to determine whether pre-motivational/motivational and volitional alcohol factors differentiated those in the control, ideation and enaction groups. Holm’s [52] sequential Bonferroni correction was used to control for multiple comparisons. All analyses included age, gender and depressive symptoms as covariates to control for possible group differences in mood and demography. Volitional phase alcohol factors which differentiated those in the ideation and enaction groups in univariate models were subsequently tested multivariately to determine their unique associations with self-harm enaction relative to ideation and control groups.

## Results

Of the 1546 participants in the study sample, 297 (19.21%) reported having thoughts of self-harm without acting upon them (the ‘ideation’ group), 346 (22.38%) reported acts of self-harm (the ‘enaction’ group) and 897 (58.02%) reported no history of thoughts or acts (the ‘control’ group). Six individuals did not respond to questions on self-harm and could not be allocated to a group. Those in the enaction group were more likely to be female (78%) than those in the ideation (68%; OR = 1.71, 95% CI = 1.20–2.43, *p* = 0.003) and control (68%; OR = 1.73, 95% CI = 1.29–2.32, *p* <  0.001) groups. Those in the enaction group also reported higher levels of depressive symptoms (Mean = 19.37, SE = 0.69) than those in the ideation group (Mean = 15.58, SE = 0.65; OR = 1.03, 95% CI = 1.01–1.04, *p* <  0.001), who in turn reported more depressive symptoms than those in the control group (Mean = 8.80, SE = 0.27; OR = 1.07, 95% CI = 1.06–1.09, *p* <  0.001). The enaction group tended to be younger (Mean = 29.03, SE = 0.57) than the ideation group (Mean = 34.12, SE = 0.75; OR = 0.97, 95% CI = 0.95–0.98, *p* <  0.001) who in turn were younger than the control group (Mean = 36.39, SE = 0.45; OR = 0.99, 95% CI = 0.98–1.00, *p =* 0.011).

Descriptive statistics for the pre-motivational and motivational phase factors as well as the volitional phase alcohol factors, by self-harm status, can be found in Table 1.

**Table 1:** Descriptive statistics (Mean, Standard Error^a^) for pre-motivational/motivational and volitional phase alcohol factors

Pre-motivational/ Motivational factors	No self-harm ideation or enaction	Self-harm ideation	Self-harm enaction
Perceived stress	5.03 (0.10)	7.03 (0.19)	7.92 (0.18)
Support	7.82 (0.05)	7.05 (0.11)	6.71 (0.10)
NMR expectancies	108.59 (0.53)	95.85 (1.00)	91.52 (1.08)
Optimism	3.77 (0.28)	3.40 (0.05)	3.21 (0.05)
Volitional phase alcohol factors			
Heavy Drinking Frequency	1.33 (0.03)	1.27 (0.06)	1.65 (0.05)
Negative Urgency-A	1.77 (0.02)	1.93 (0.05)	2.34 (0.04)
CEOA: Sociability	2.83 (0.02)	2.94 (0.04)	3.06 (0.03)
CEOA: Tension Reduction	2.52 (0.02)	2.59 (0.04)	2.59 (0.04)
CEOA: Liquid Courage	2.10 (0.03)	2.26 (0.04)	2.42 (0.04)
CEOA: Cognitive & Behavioural Impairment	2.44 (0.02)	2.65 (0.04)	2.73 (0.03)
CEOA: Self-Perception	1.63 (0.02)	1.87 (0.04)	2.00 (0.03)
CEOA: Risk & Aggression	1.81 (0.02)	1.98 (0.04)	2.14 (0.04)
Alcohol Expectancy: Self-Harm	1.33 (0.01)	1.52 (0.03)	1.68 (0.03)

No self-harm ideation or enaction: *n* = 897, Self-harm ideation: *n* = 297, Self-harm enaction: *n* = 346

NMR expectancies: Generalised Expectancies for Negative Mood Regulation scale, CEOA: Comprehensive Effects of Alcohol scale

^a^Alternative measures of variance (i.e. standard deviation) cannot be generated for multiple imputed data using the statistical package used in the analysis

As expected univariate analyses (Table 2) indicated that those with a history of self-harm (those in ideation or enaction groups) reported significantly greater stress, lower perceived social support and more limited beliefs in their ability to address negative mood states compared to controls. Those with a history of self-harm enaction reported significantly lower optimism than those in the control group, but those in the control and ideation groups did not differ. Also as expected none of the pre-motivational/motivational phase factors differentiated between those in the ideation and enaction groups. In contrast the enaction and ideation groups did differ significantly from the control group and one another in the expected direction on several volitional phase alcohol factors (Table 3): the enaction group reported more frequent heavy drinking, higher alcohol-related negative urgency, as well as stronger expectancies that alcohol will lead to negative self-perceptions (Self-Perception) and markers of self-harm risk (Self-Harm alcohol expectancies). Expectancies that alcohol will lead to impaired functioning (Cognitive and Behavioural Impairment), enhance confidence and bravery (i.e. Liquid Courage) and risky and aggressive behaviour (Risk and Aggression) differed significantly across groups but not between the ideation and enaction groups.

**Table 2:** Univariate multinomial logistic regression analyses of the association between self-harm status and pre-motivational/motivational phase factors (adjusted for age, gender, depressive symptoms)

	Self-harm status	OR	CI	P
Pre-motivational/Motivational factors				
*Perceived stress*				
No self-harm enaction or ideation	Self-harm ideation	1.10	1.04–1.17	0.002
No self-harm enaction or ideation	Self-harm enaction	1.12	1.06–1.19	< 0.001
Self-harm ideation	Self-harm enaction	1.02	0.95–1.09	0.584
*Support*				
No self-harm enaction or ideation	Self-harm ideation	0.90	0.82–0.98	0.019
No self-harm enaction or ideation	Self-harm enaction	0.84	0.77–0.92	< 0.001
Self-harm ideation	Self-harm enaction	0.94	0.85–1.04	0.208
*NMR expectancies*				
No self-harm enaction or ideation	Self-harm ideation	0.97	0.96–0.98	< 0.001
No self-harm enaction or ideation	Self-harm enaction	0.97	0.96–0.98	< 0.001
Self-harm ideation	Self-harm enaction	1.00	0.99–1.01	0.441
*Optimism*				
No self-harm enaction or ideation	Self-harm ideation	0.83	0.69–1.00	0.053
No self-harm enaction or ideation	Self-harm enaction	0.75	0.62–0.90	0.002
Self-harm ideation	Self-harm enaction	0.90	0.73–1.10	0.299

*OR* Odds ratio, *CI* 95% Confidence intervals

All statistically significant comparisons remain statistically significant after applying Holm’s sequential Bonferroni correction

NMR expectancies: Generalised Expectancies for Negative Mood Regulation scale

**Table 3:** Univariate multinomial logistic regression analyses of the association between self-harm status and volitional phase alcohol factors (adjusted for age, gender, depressive symptoms)

	Self-harm status	OR	CI	P
Volitional phase alcohol factors				
*Negative Urgency-A*				
No self-harm enaction or ideation	Self-harm ideation	1.11	0.91–1.35	0.314
No self-harm enaction or ideation	Self-harm enaction	1.93	1.60–2.33	< 0.001
Self-harm ideation	Self-harm enaction	1.74	1.41–2.16	< 0.001
*Heavy Drinking Frequency*				
No self-harm enaction or ideation	Self-harm ideation	0.90	0.78–1.03	0.133
No self-harm enaction or ideation	Self-harm enaction	1.31	1.13–1.51	< 0.001
Self-harm ideation	Self-harm enaction	1.46	1.24–1.72	< 0.001
*Alcohol expectancies*				
*CEOA: Sociability*				
No self-harm enaction or ideation	Self-harm ideation	1.08	0.87–1.32	0.494
No self-harm enaction or ideation	Self-harm enaction	1.21	0.97–1.50	0.094
Self-harm ideation	Self-harm enaction	1.12	0.87–1.44	0.371
*CEOA: Tension Reduction*				
No self-harm enaction or ideation	Self-harm ideation	1.05	0.86–1.28	0.642
No self-harm enaction or ideation	Self-harm enaction	1.05	0.86–1.29	0.637
Self-harm ideation	Self-harm enaction	1.00	0.80–1.26	0.991
*CEOA: Liquid Courage*				
No self-harm enaction or ideation	Self-harm ideation	1.14	0.94–1.38	0.186
No self-harm enaction or ideation	Self-harm enaction	1.34	1.10–1.63	0.004
Self-harm ideation	Self-harm enaction	1.18	0.94–1.47	0.151
*CEOA: Cognitive & Behavioural Impairment*				
No self-harm enaction or ideation	Self-harm ideation	1.34	1.08–1.65	0.007
No self-harm enaction or ideation	Self-harm enaction	1.49	1.19–1.85	< 0.001
Self-harm ideation	Self-harm enaction	1.11	0.86–1.43	0.415
*CEOA: Self-Perception*				
No self-harm enaction or ideation	Self-harm ideation	1.52	1.20–1.92	< 0.001
No self-harm enaction or ideation	Self-harm enaction	2.02	1.60–2.55	< 0.001
Self-harm ideation	Self-harm enaction	1.33	1.03–1.72	0.03
*CEOA: Risk & Aggression*				
No self-harm enaction or ideation	Self-harm ideation	1.22	0.98–1.51	0.074
No self-harm enaction or ideation	Self-harm enaction	1.51	1.22–1.87	< 0.001
Self-harm ideation	Self-harm enaction	1.24	0.97–1.58	0.082
*Alcohol Expectancy: Self-Harm*				
No self-harm enaction or ideation	Self-harm ideation	1.85	1.34–2.56	< 0.001
No self-harm enaction or ideation	Self-harm enaction	3.04	2.22–4.17	< 0.001
Self-harm ideation	Self-harm enaction	1.64	1.18–2.30	0.004

*OR* Odds ratio, *CI* 95% Confidence intervals

All statistically significant comparisons remain statistically significant after applying Holm’s sequential Bonferroni correction

*CEOA* Comprehensive Effects of Alcohol scale

To determine their relative contribution to self-harm enaction the factors which distinguished between the ideation and enaction groups in the univariate analysis were entered into a multivariate multinomial logistic regression model: younger age (OR = 0.97, 95% CI = 0.96–0.99, *p* <  0.001), female gender (OR = 1.81, 95% CI = 1.24–2.66, *p* = 0.002), more frequent heavy drinking (OR = 1.32, 95% CI = 1.11–1.58, *p* = 0.002) and alcohol-related negative urgency (OR = 1.47, 95% CI = 1.15–1.88, *p* = 0.002) differentiated those in the ideation and enaction groups. Self-Perception alcohol expectancies (OR = 0.85, 95% CI = 0.58–1.23, *p* = 0.382), Self-Harm alcohol expectancies (OR = 1.61, 95% CI = 1.00–2.59, *p* = 0.051) and depressive symptoms (OR = 1.01, 95% CI = 1.00–1.03, *p* = 0.056) did not differentiate those in the enaction and ideation groups in the multivariate model. Those in the enaction group also differed from those in the control group on each factor, apart from Self-Perception alcohol expectancies, and frequency of heavy drinking, following adjustment for multiple comparisons (see Additional file 1: Table S4).

## Discussion

We have identified volitional phase alcohol factors associated with a history of self-harm enaction in a healthy community sample of the Scottish population. Increasing frequencies of drinking to intoxication and a greater tendency to engage in rash actions in order to reduce negative affect when drinking (i.e., alcohol-related negative urgency) were independently associated with self-harm enaction. Expecting alcohol to lead to outcomes consistent with increasing risk of self-harm followed a similar pattern of differentiating those in the ideation and enaction groups in univariate analyses only. Studied within an ideation-to-action framework [30, 32] these relationships were specified a priori as potential paths towards self-harm enaction and were observed independently of depressive symptoms and demographic characteristics. This represents an important advance in current knowledge by focusing attention on several potential alcohol-related mechanisms of self-harm enaction rather than the presence or absence of underlying alcohol use disorder (e.g., [9, 10]). Also as expected perceived stress, lower social support and more limited expectancies for negative mood regulation differed between those with a history of self-harm thoughts and acts, but did not differentiate those with a history of thinking about self-harm from those with a history of self-harm acts. Although we were surprised that more of the sample reported self-harm acts than thoughts of self-harm, this may simply reflect our self-harm classification, which combines suicidal and non-suicidal thoughts/acts and may be driven by a relatively high prevalence of non-suicidal self-harm in this population.

Whether the volitional phase alcohol factors described here have a causal role in self-harm enaction cannot be inferred from the cross-sectional study design and retrospective accounts of self-harm histories and alcohol-related thoughts and behaviours recorded in this study. However, we have identified plausible mechanisms by which volitional phase alcohol factors may lead to self-harm enaction and propose that these should be examined further in prospective studies to understand their role in any transition from thinking about self-harm to self-harm acts. These include intoxication exacerbating or leading to an escalation in emotional distress combined with a restricted ability to formulate adaptive coping strategies, a behavioural tendency to act rashly in response to negative affect when drinking, and behaving in accordance with one’s expectations that alcohol will lead to outcomes reflecting increasing risk for self-harm. Of particular concern is that features of these volitional phase alcohol factors may be self-reinforcing. To the extent that engaging in rash actions in response to distressing thoughts or emotions provides immediate short-term relief, and the outcomes of drinking are consistent with one’s expectancies, they may be reinforcing and establish or strengthen similar alcohol-related responses in future.

Because a sizeable minority of the Scottish adult population (35% men, 17% women) regularly drink at hazardous or harmful levels [33], and lifetime prevalence of self-harm is also substantial [53], the potential contribution of research on volitional phase alcohol factors to prevention and management of self-harm may be significant. Importantly, none of the volitional phase alcohol factors associated with self-harm enaction are a feature of clinical disorder and high or low levels of each may be considered a point on a continuum of normal functioning. Although standard assessments for alcohol use disorders which are recommended for use in routine practice may capture the frequency of intoxication (e.g., [43, 54]), these are unlikely to include questions sensitive to capturing risk from other volitional phase alcohol factors. To the extent that further research provides robust evidence that volitional alcohol factors are associated with a transition from self-harm thoughts to self-harm acts, then assessment of risk for alcohol-related self-harm enaction may need to go beyond standard questions on quantity and frequency of alcohol use to assess behavioural tendencies and expectations when drinking alcohol.

Several additional comments on these volitional alcohol factors are needed. Two of the potential mechanisms of alcohol-related self-harm enaction we propose posit emotional distress as a contributing factor, yet negative affect was assessed only as part of the alcohol-related negative urgency measure. The extent to which emotional distress has a critical role to play in the relationship between intoxication and self-harm enaction therefore requires further investigation. Given limited prior empirical research on the role of alcohol expectancies as potential mechanisms of self-harm enaction, we investigated multiple common outcome expectancy domains. Only self-harm alcohol expectancies approached the conventional level of statistical significance (i.e. *p* = .051) when considered alongside other volitional phase alcohol factors. While self-harm expectancies were based on a novel set of items, and have therefore not previously been psychometrically tested, we used a standard alcohol expectancy item format and based item content on contemporary theories of self-harm [30, 49]. Furthermore, the ability of the novel self-harm alcohol expectancy measure to differentiate those with thoughts of self-harm from those who have acted on their thoughts in the univariate analyses suggests there is potential utility of this measure.

There are a number of limitations to this study. Counter-to-our hypothesis, optimism did not differ between those with and without a history of self-harm. We can provide no definitive explanation, but note that relevant studies reporting lower levels of optimism in those with a history of self-harm have tended to utilise more comprehensive multi-item assessments of optimism rather than the single item in the present study (e.g., [31]). Further possible limitations stem from the use of a cross-sectional study design, and the measures of pre-motivational/motivational, volitional alcohol factors and self-harm used in this study. Specifically, some pre-motivational/motivational and volitional alcohol factors were assessed as relatively stable phenomena, while others assessed a recent specific period. In combination with the cross-sectional study design, it cannot be claimed that these measurements necessarily align or precede the development or transition of self-harm thoughts and acts. Furthermore, a number of alternative interpretations for these findings cannot be ruled out, including the possibility that volitional alcohol factors may reflect a maladaptive coping strategy which emerges after rather than prior to a self-harm act or that both self-harm and volitional alcohol factors are a consequence of underlying problems in self-regulation. Furthermore, the observed associations between volitional phase alcohol factors and self-harm enaction may also reflect historical or residual risk due to past alcohol-related behaviour and problems. Recent studies have sought to parse acute effects of alcohol on risk of suicide attempt from past behaviour utilising case-crossover designs, in which any alcohol-related features of an index suicide attempt are compared with a recent reference period from the same individual’s past (e.g., [55]). Much can be gained from such designs, in which an individual or case also acts as a control for their own behaviour, but practical constraints mean there is often a focus on smaller samples of acutely suicidal patients. In contrast, the present research reports findings from a larger generally healthy population which are relevant to those across a spectrum of self-harm thoughts and acts. We would argue that a range of observational study designs is needed to provide converging evidence of the role of volitional alcohol factors.

## Conclusion

Management of self-harm risk requires better understanding of the alcohol-related mechanisms of self-harm enaction. Volitional alcohol factors may play a role in governing the translation of self-harm thoughts into self-harm acts.

## Supplementary information

**Additional file 1: Figure S1: **Health, Lifestyle and Wellbeing Study participants. **Table S4.** Multivariate multinomial logistic regression analyses of the association between self-harm status and volitional phase alcohol factors.

## Data Availability

The dataset supporting the conclusion of the current study is available from the corresponding author on reasonable request.

## Abbreviations

95% CI: 95% confidence interval
AUD: Alcohol-use disorder
CEOA: Comprehensive Effects of Alcohol Questionnaire
CESD-R: Centre for Epidemiologic Studies Depression Scale-Revised
IMV model: Integrated Motivational-Volitional model
MAR: Missing at random
Negative Urgency-A: Negative Urgency subscale of the UPPS Impulsive Behavior Scale (modified for this study)
NMR: Generalised Expectancies for Negative Mood Regulation Scale
OR: Odds ratio
PSS-S: Perceived Stress Scale Short Form
SD/E: Standard deviation/error
URL: Uniform resource locator

## Footnotes

1 Scale internal consistency statistics (i.e. Cronbach’s α) reported throughout this article are based on complete cases.

2 The CEOA subscale ‘Sexuality’ was not included on the basis of limited relevance.
