# Supplementary material for: Differentiating adults who think about self-harm from those who engage in self-harm: the role of volitional alcohol factors
Source: BMC Psychiatry. 2019 Oct 28;19:319. doi: 10.1186/s12888-019-2292-3 (PMC6816185; doi:10.1186/s12888-019-2292-3)
Supplement: Supplementary file 1 — Additional file 1: Figure S1: Health, Lifestyle and Wellbeing Study participants. Table S4. Multivariate multinomial logistic regression analyses of the association between self-harm status and volitional phase alcohol factors. [file 12888_2019_2292_MOESM1_ESM.docx]

Excluded (Total = 19)

- Did not consent n = 7
- Withdrew n = 1
- Duplicate responses n = 11

Did not provide minimum dataset (Total = 759)

- >50% missing data item level n = 592
- >10% missing data multi-item scales n = 167

Data available for analysis (Total = 1546)

Observed Missing (%) Multiple Imputation

- Age n = 1519 1.75 yes n = 1546
- Gender n = 1539 0.45 no n = 1539
- CESD-R (depression) n = 1486 3.88 yes n = 1546
- PSS-4 (stress) n = 1543 0.19 yes n = 1546
- Social support n = 1540 0.39 yes n = 1546
- Optimism n = 1545 0.06 yes n = 1546
- NMR expectancies n = 1379 10.9 yes n = 1546
- Alcohol expectancies* n = 1511-1537 0.58-2.26 yes n = 1546
- Negative Urgency-A n = 1523 1.49 yes n = 1546
- Self-harm n = 1540 0.4 no n =1540

No ideation/enaction n = 897

Ideation n = 297

Enaction n = 346

*ns vary across subscales

Responded to invitation and accessed study materials (Total = 2324)

- Online n = 2322
- Postal n = 2

Adults (>17years) resident in Scotland invited to participate via large commercial and local authority employers, university registers, online community groups and forums.

Figure S1: Health, Lifestyle and Wellbeing Study participants

Table S4. Multivariate multinomial logistic regression analyses of the association between self-harm status and volitional phase alcohol factors (adjusted for age, gender, depressive symptoms)

|  | **Self-harm status** | **OR** | **CI** | **P** |
| --- | --- | --- | --- | --- |
| *Age* | |  |  |  |
| No self-harm enaction or ideation | Self-harm ideation | 0.99 | 0.98-1.00 | 0.132 |
| No self-harm enaction or ideation | Self-harm enaction | 0.96 | 0.95-0.98 | < 0.001 |
| Self-harm ideation | Self-harm enaction | 0.97 | 0.96-0.99 | <0.001 |
| *Gender* | |  |  |  |
| No self-harm enaction or ideation | Self-harm ideation | 0.95 | 0.70-1.28 | 0.718 |
| No self-harm enaction or ideation | Self-harm enaction | 1.71 | 1.21-2.42 | 0.002 |
| Self-harm ideation | Self-harm enaction | 1.81 | 1.24-2.66 | 0.002 |
| *Depressive symptoms* | |  |  |  |
| No self-harm enaction or ideation | Self-harm ideation | 1.06 | 1.05-1.08 | <0.001 |
| No self-harm enaction or ideation | Self-harm enaction | 1.08 | 1.06-1.09 | < 0.001 |
| Self-harm ideation | Self-harm enaction | 1.01 | 1.00-1.03 | 0.056 |
| **Volitional phase alcohol factors** | |  |  |  |
| *Negative Urgency-A* | |  |  |  |
| No self-harm enaction or ideation | Self-harm ideation | 1.02 | 0.82-1.28 | 0.837 |
| No self-harm enaction or ideation | Self-harm enaction | 1.51 | 1.21-1.87 | < 0.001 |
| Self-harm ideation | Self-harm enaction | 1.47 | 1.15-1.88 | 0.002 |
|  |  |  |  |  |
|  |  |  |  |  |
|  |  |  |  |  |
|  |  |  |  |  |
|  | **Self-harm status** | **OR** | **CI** | **P** |
| *Heavy Drinking Frequency* |  |  |  |  |
| No self-harm enaction or ideation | Self-harm ideation | 0.90 | 0.77-1.05 | 0.176 |
| No self-harm enaction or ideation | Self-harm enaction | 1.19 | 1.02-1.39 | 0.030^NS^ |
| Self-harm ideation | Self-harm enaction | 1.32 | 1.11-1.58 | 0.002 |
| *Alcohol expectancies* |  |  |  |  |
| *CEOA: Self-Perception* |  |  |  |  |
| No self-harm enaction or ideation | Self-harm ideation | 1.28 | 0.92-1.78 | 0.150 |
| No self-harm enaction or ideation | Self-harm enaction | 1.08 | 0.77-1.51 | 0.648 |
| Self-harm ideation | Self-harm enaction | 0.85 | 0.58-1.23 | 0.382 |
| *Alcohol Expectancy: Self-Harm* |  |  |  |  |
| No self-harm enaction or ideation | Self-harm ideation | 1.45 | 0.91-2.27 | 0.111 |
| No self-harm enaction or ideation | Self-harm enaction | 2.33 | 1.49-3.62 | <0.001 |
| Self-harm ideation | Self-harm enaction | 1.61 | 1.00-2.59 | 0.051 |

OR: Odds ratio, CI: 95% Confidence intervals

^NS^ No longer statistically significant after applying Holm’s sequential Bonferroni correction.

CEOA: Comprehensive Effects of Alcohol scale
